# Usability and User Experience of a Digital Platform Prototype (Healthy Bone) to Promote Pharmacological and Nonpharmacological Treatment in Patients With Osteoporosis: Mixed Methods Study

**DOI:** 10.2196/72468

**Published:** 2025-11-07

**Authors:** Anabela Barcelos, Alexandre Moniz, Ana Rita Henriques, Carolina Mazeda, Elsa Frazão Mateus, Ana Machado, Cátia F Gonçalves, Maiara Moreto, Mafalda F Almeida, Nuno Mendonça, Daniela Costa, Marta M Marques, Pedro Domingos, Yoann Nesme, Helena Canhão, Sónia Dias, Ana Maria Rodrigues

**Affiliations:** 1 Rheumatology Department, Unidade Local de Saúde de Aveiro, Rua Artur Ravara, 35, Aveiro, Portugal, 351 234 378 300; 2 EpiDoc Unit, NOVA Medical School, Comprehensive Health Research Center (CHRC), Universidade NOVA de Lisboa, Lisboa, Portugal; 3 NOVA National School of Public Health, Public Health Research Centre, Comprehensive Health Research Center (CHRC), Universidade NOVA de Lisboa, Lisboa, Portugal; 4 Liga Portuguesa Contra as Doenças Reumáticas, Lisboa, Portugal; 5 OrangeBird Lda, Lisboa, Portugal

**Keywords:** eHealth, information and communication technologies, osteoporosis-related fractures, older adults, osteoporosis, usability

## Abstract

**Background:**

Osteoporosis-related fractures significantly impact older adults, often leading to disability and even premature death. While pharmacological and nonpharmacological interventions are widely recommended for managing osteoporosis, adherence to these interventions remains low. To address this challenge, we developed the Healthy Bone digital platform (desktop, mobile app, and smart TV internet-based) for use in clinical settings to improve disease management and treatment adherence. It integrates a multimedia health-related behavioral change program with a patient monitoring and management system.

**Objective:**

This study aimed to evaluate the usability and user experience of the desktop version of the Healthy Bone digital platform prototype from the patients’ perspective. The findings will provide valuable insights into optimizing the digital platform and enhancing its functionality.

**Methods:**

A mixed-methods study was conducted. During usability testing, patients completed tasks simulating real-world use of the platform while using a Think-Aloud approach. After each task, participants filled out an After Scenario Questionnaire to assess task satisfaction. Subsequently, participants completed the System Usability Scale (SUS) and the eHealth Usability Benchmarking Instrument (HUBBI) to measure usability quantitatively. Following this, semistructured interviews were conducted to explore participants’ experiences with the platform in greater depth. Descriptive statistics were used for quantitative analysis. Qualitative data analysis involved a combined deductive and inductive approach, ensuring a comprehensive evaluation of the platform’s usability and user experience. Deductive content analysis was guided by an ontology of eHealth usability components, while thematic analysis adhered to Braun and Clarke’s method to identify emerging themes.

**Results:**

Seven participants evaluated the digital platform, reporting high usability with a mean overall SUS score of 87.1 (SD 13.3). Similarly, good usability was reported across all categories of the HUBBI, except for the guidance and support category, which presented moderate levels of usability (mean 3.3, SD 1.1). Patients reported high levels of task satisfaction and identified 24 unique usability issues, predominantly related to the basic system performance, interface design, and navigation and structure categories of the eHealth usability ontology. Overall, patients had positive perceptions and acceptability of the digital platform, highlighting its simplicity, accessibility, utility, and potential to empower those with osteoporosis. Barriers to usage included limited skills, lack of suitable equipment, and time, while facilitators included motivation for behavior change, health benefits, and the decrease of potential inequalities.

**Conclusions:**

This study provided valuable insights into the usability and user experience of the desktop version of the Healthy Bone digital platform prototype. These findings will play a key role in optimizing the platform to ensure it is effectively tailored to the needs of the target population. This platform adds an understanding of how various information and communication technology tools can support and benefit large numbers of osteoporosis patients in society.

## Introduction

### Background

Worldwide, osteoporosis causes more than 8.9 million fractures annually, a number that is estimated to rise to around 200 million over the next 50 years [1]. Estimates have shown that the direct health care costs resulting from fractures in postmenopausal or over 50-year-old women are €57‐74 (US$62-US$80) million per year [2,3]. Across Europe, the number of fracture-related deaths is comparable to or higher than that of some of the most common causes of death, such as diabetes, lung cancer, and chronic lower respiratory tract diseases [4]. Osteoporosis-related fractures constitute a major public health challenge and result in increased disability, morbidity, and mortality, especially among older adults [5], imposing a huge and growing economic burden on health care systems [5,6].

Given the urgency of addressing this issue, there is a need to promote appropriate treatment of osteoporosis and adopt measures to prevent the occurrence of first and recurrent osteoporosis-related fractures. To achieve this, pharmacological and nonpharmacological (eg, good nutrition, exercise and physical activity, and avoidance of harmful behaviors, such as smoking and alcohol consumption) management strategies have been widely recommended [7-9]. However, a considerable treatment gap is currently characterized by low screening and treatment adherence rates [5,10,11]. For example, poor adherence to exercise (36%‐55%) and physical activity (14%‐58%) [12] and low to moderate adherence to pharmacological treatments (40%‐70%) [13,14] by patients with osteoporosis have been previously reported. This highlights the need for developing and implementing self-management interventions focused on behavior change support, especially in ensuring patients adopt pharmacological and nonpharmacological osteoporosis management strategies.

The emergence of new information and communication technologies (ICTs) offers promising tools to address barriers to treatment adherence [15]. The rapid development of computer-based technologies—coupled with the widespread availability of the internet and mobile and TV apps—has led to the rise of the concepts of “eHealth” and “mHealth” [16-18]. These digital solutions have the potential to foster positive health behavior change, support healthier lifestyles, and assist in the diagnosis and treatment of diseases [19]. As a growing interdisciplinary field, eHealth and mHealth sit at the intersection of medical informatics, public health, and business. It refers to health services and information that are delivered or enhanced through the internet and related technologies [19-21]. Computer-based technologies can support health care professionals in remotely monitoring patients and assisting with daily activities, such as medication reminders and education about personal habits and behavioral patterns. These tools enable the customization of interventions, particularly in the management of chronic diseases [22-24]. Several studies have highlighted the positive impact of such technologies on enhancing patient competence [24-26]. Informed patients are more likely to adopt healthy behaviors and effectively manage their conditions [27,28].

With the aim of promoting health care, a growing number of studies have explored the use of mobile apps across different medical domains, showing promising results [26,27,29-31]. These apps allow patients to access relevant health information anytime and anywhere, empowering them in their self-care journey.

In the context of osteoporosis, mHealth apps have emerged as valuable tools for both patients and health care providers [27,32,33]. They can enhance disease management by providing broad, cost-effective, and accessible support to a large population [34]. Effective self-management apps for osteoporosis should include comprehensive educational content, covering the nature of the disease [35], dietary and medication guidance [36-38], physical activity recommendations [39], and strategies to prevent falls and fractures [40].

The idea of individual responsibility in the self-management of osteoporosis has gained momentum in recent decades, and mHealth apps can play a crucial role in supporting this shift. However, despite the growing number of apps aimed at osteoporosis self-management, many lack clinically validated evidence of effectiveness [33,41]. According to Alhussein et al [33], approximately 75% of the mHealth apps reviewed in their systematic review and meta-analysis had not undergone premarket prospective multicenter randomized controlled trials (RCTs), likely due to the high costs and time-consuming nature of patient recruitment [42]. In addition, many apps failed to demonstrate their usability and acceptability among users [33,43].

More recently, Bendtsen et al [41] assessed the eHealth literacy of Danish osteoporosis patients using the app “My Bones.” Their findings indicated that the app had good usability and the potential to reduce general practitioner visits, improve health outcomes, and serve as a valuable complement to standard health care and social services.

A successful mHealth app should go beyond promoting behaviors such as healthy eating, physical activity, or medication adherence. It must foster a genuine sense of individual health responsibility and support the self-management of osteoporosis in a sustainable and meaningful way.

An international consortium coordinated by our research group has successfully developed and evaluated a novel digital platform, the Saúde.come TV app [44]. This interactive multimodule, customizable app delivered a home-based program to promote healthy behaviors among older adults with food insecurity. The results showed improvement in dietary behaviors, physical activity, and a reduction in participants’ BMI [31]. The success of this TV platform has reinforced the development of similar platforms, namely for patients with osteoporosis with or without prevalent osteoporosis-related fractures. Therefore, the prototype for the Healthy Bone digital platform (desktop, mobile app, and smart TV internet-based) was developed as a web-based multimodule and interactive application containing a combination of patient monitoring and management systems with a multimedia health-related behavioral change program. It includes education strategies, patient empowerment, self-management reminders, and prompts designed to improve disease management in a clinical setting and increase long-term adoption of nonpharmacological and pharmacological osteoporosis treatment.

To ensure that the Healthy Bone digital platform is satisfactory, well-adapted to its intended users, and successfully implemented, it is crucial to assess its usability. Usability is defined as “the extent to which specified users can use a system, product, or service to achieve specified goals with effectiveness, efficiency, and satisfaction in a specified context of use” [45]. The usability assessment is considered an essential step in developing eHealth innovations, as it improves interfaces and enhances user interaction [46]. The Healthy Bone digital platform prototype was designed to facilitate and promote behavior changes. To verify if this goal is achieved, it is important to assess the usability and user experience of the Healthy Bone digital platform.

### Objective

This study aimed to evaluate the system usability and user experience of the desktop version of the Healthy Bone digital platform prototype from the patients’ perspective. The information gathered will inform the optimization of this digital platform.

## Methods

### Study Design

A mixed-methods study used a convergent approach, combining quantitative data from self-reported questionnaires and qualitative data from Think-Aloud sessions and semistructured interviews. To enhance the transparency and reporting, this study adhered to the Good Reporting of a Mixed Methods Study (GRAMMS) checklist [47] (Checklist 1).

### Participants and Recruitment

Participants were recruited at the rheumatology department of Centro Hospitalar do Baixo Vouga, Aveiro, Portugal. The study sample included patients with osteoporosis recruited consecutively from the Osteoporosis Clinic (representing the potential end users of the digital platform). A purposive sample strategy was used to ensure the inclusion of participants who might provide relevant, valuable, and detailed feedback. Participants were included if they fulfilled the following criteria: (1) community-dwelling older adults (≥65 years old); (2) diagnosis of osteoporosis based on either the *t* score criterion (*t*≤-2.5 at the lumbar spine, or hip neck); (3) basic knowledge about the use of digital solutions; (4) proficiency in Portuguese; and (5) able and willing to give written informed consent and comply with the requirements of the study. Participants were excluded if they could not comply with the study procedures due to hearing or visual loss, significant cognitive impairment, and illiteracy. A minimum sample size of 5 participants was set for usability testing, as this is sufficient to identify over 80% of usability issues [48].

### Healthy Bone Digital Platform

The Healthy Bone digital platform prototype, accessible via desktop, mobile app, and smart TV, was developed as a web-based, multimodule, and interactive application. It integrates a multimedia health-related behavioral change program with a patient monitoring and management system. This prototype was developed based on a previous app developed by our team, the Saúde.Come TV app [31,44]. The Healthy Bone platform integrates educational strategies, patient empowerment tools, self-management reminders, and prompts designed to improve disease management and promote long-term adherence to pharmacological and nonpharmacological osteoporosis treatments. The Healthy Bone digital platform was designed to be used in a clinical setting to improve the long-term management of patients who live with osteoporosis with or without osteoporosis-related fractures. This platform aims to deliver comprehensive and user-centered support for managing osteoporosis.

### Healthy Bone System Architecture

**Figure 1.**
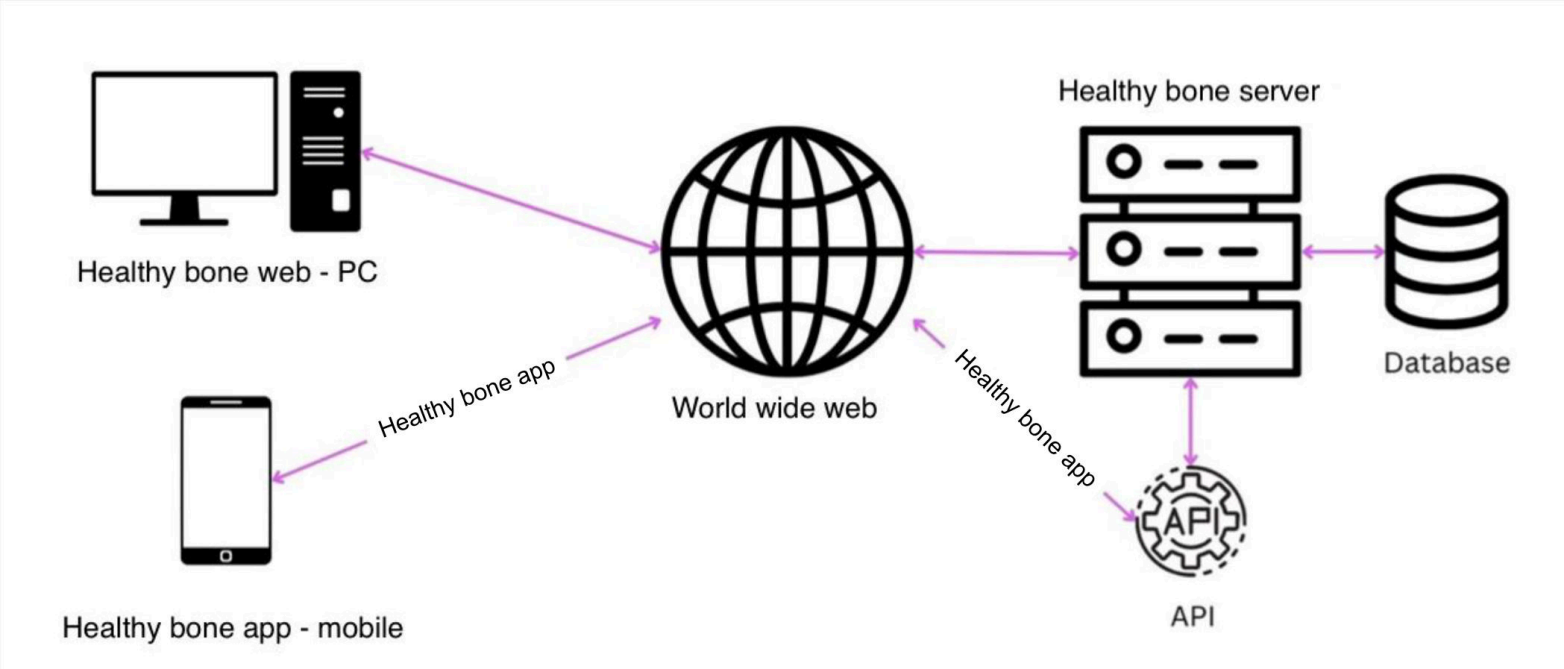
The Healthy Bone system architecture. API: Application Programming Interface.

The Healthy Bone digital platform was designed by the principal investigator (AMR) and custom-developed by the Orange Bird company. Coinvestigators contributed to content development (HC, AB, AM, NM, and MMM) with guidance on the visual display provided by experienced app developers. EFM (coinvestigator and patient research partner) contributed to content development through several meetings with the principal investigator. Other patients were also involved in structured interviews. The technical architecture of the Healthy Bone platform (Figure 1) is based on the open-source Drupal Content Management System (CMS). The CMS works as the main repository for content (such as users, streaming videos, and questionnaires) and provides the web interface of the digital solution. An Application Programming Interface (API) was developed so that the CMS (backend) can interact with other interfaces, such as mobile apps. Additional off-the-shelf and custom modules were deployed on top of the base CMS. The development was implemented to allow further features to be added as requested.

**Figure 2.**
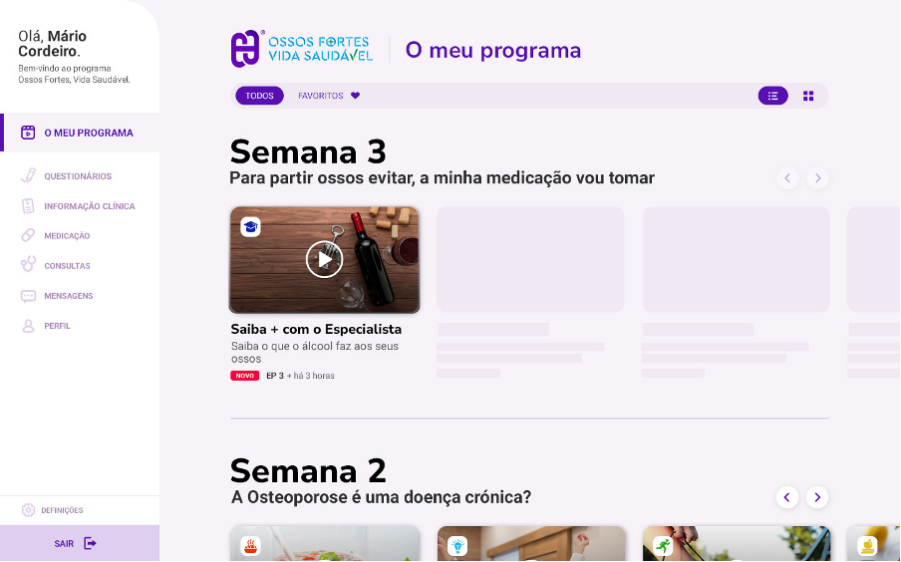
Screenshot of the Healthy Bone platform main page (My Program).

The Healthy Bone digital platform prototype has several pages, each offering different content and features (Figures2  3). On the main page, titled “My Program,” patients can access multimedia related to osteoporosis care, including videos they can watch, like or dislike, and mark as “Favorites.” The “Questionnaires” page allows participants to complete weekly questionnaires on topics covered in the daily multimedia content. On this page, they can also fill out patient-reported outcome measures, such as the Health Assessment Questionnaire (HAQ) [49], the Self-Mini Nutritional Assessment [50], or the short form of the International Physical Activity Questionnaire [51]. The “Clinical Information” page displays graphical representations of the participants’ responses to the questionnaire. On the “Medication” page, participants can track their medication and rheumatology appointments on the “Appointments” page. The “Messages” page offers a dedicated chat feature for direct communication between participants and medical doctors. Finally, the “Profile” page displays participants’ personal information and their progress through the program, including details such as their current phase, the number of videos they have watched, and the number of questionnaires they completed.

**Figure 3.**
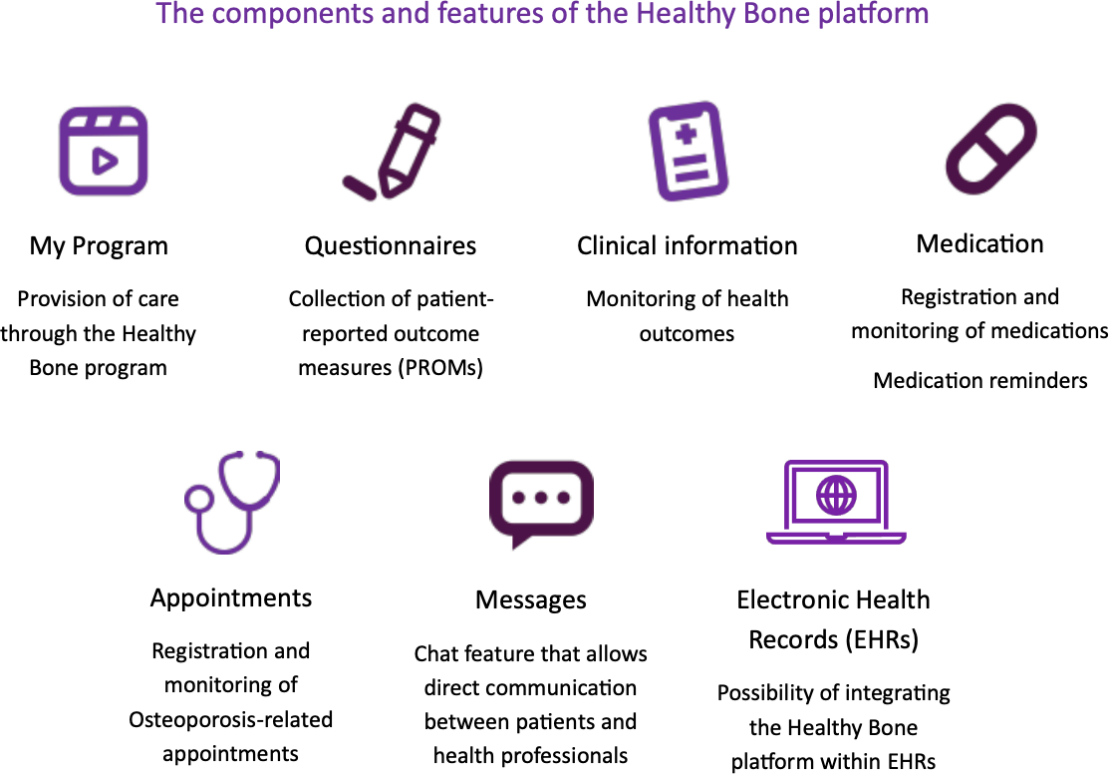
Synthesis of the platform’s several content and features.

This digital platform allows health care professionals to access patient data and monitor program adherence, potentially improving continuity of care.

### Healthy Bone Program Description

**Figure 4.**
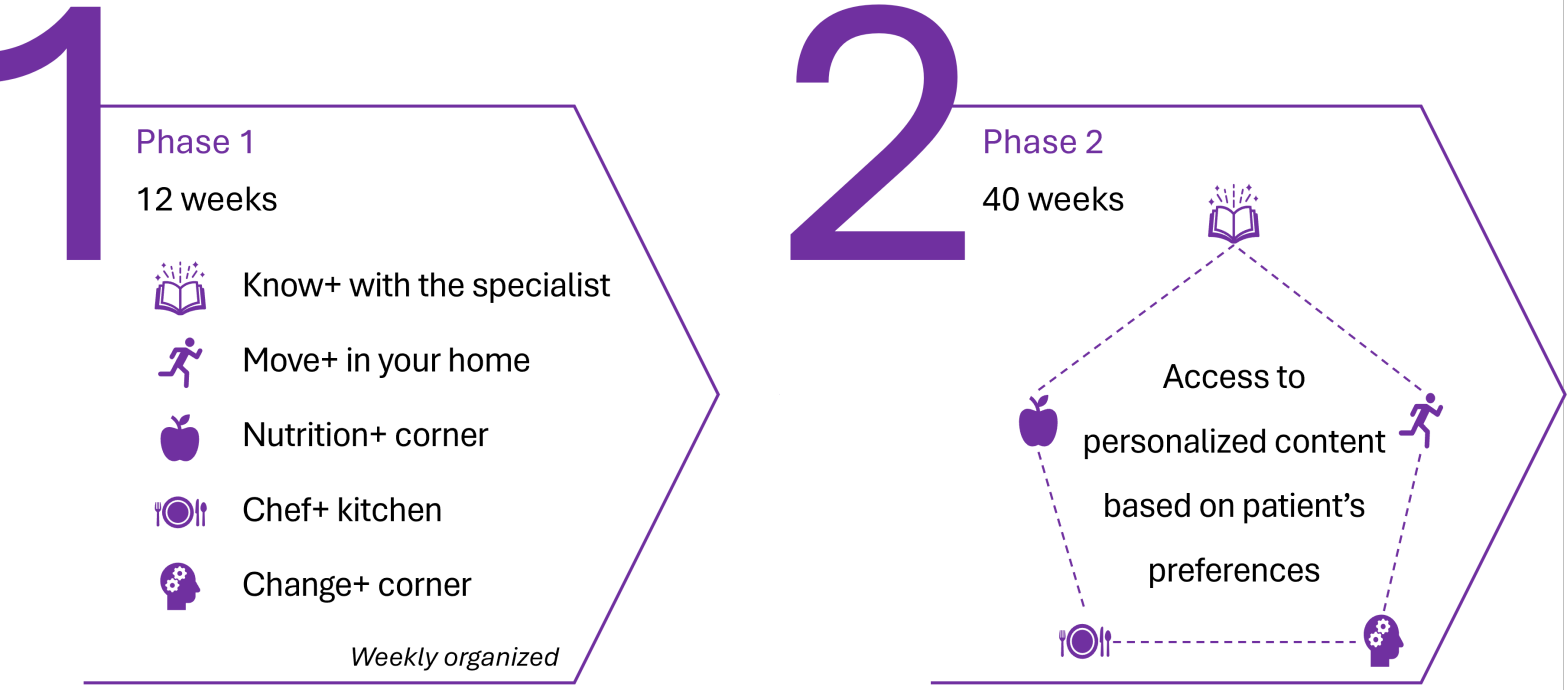
Overview of the Healthy Bone program.

The Healthy Bone program is divided into 2 phases, lasting 52 weeks (Figure 4). Phase 1 lasts for 12 weeks, is weekly organized, and is composed of original multimedia content, with one video released daily. The multimedia content includes educational content on healthy aging and bone fragility, exercise plans, nutritional advice, strategies for behavior change, and treatment reminders. Each week is composed of 5 modules: (1) Know+ with your specialist, (2) Move+ in your home, (3) Nutrition+ Corner, (4) Change+ Corner, and (5) Chef+ Kitchen. In phase 2, which occurs from Week 13 to Week 52, participants gain access to the entire library of multimedia content, now organized by themes. Additional content may be uploaded to the platform to address individual participants’ needs. Throughout the program, participants may access their clinical information, including all relevant medication, and contact their health professionals through a dedicated chat feature.

### Usability Testing

#### Data Collection

Participants were asked to complete a questionnaire that collected their sociodemographic information and clinical characteristics. Sociodemographic characteristics included age, sex, educational background, use of technological devices, and the most common tasks they perform using a computer. For clinical characterization, participants completed the European Portuguese versions of the EuroQol with 5 dimensions and 3 levels (EQ-5D-3L) [52] to assess quality of life, and the HAQ [49] to evaluate functional disability.

Usability testing (qualitative and quantitative methods) and semistructured interviews were performed to test and gather feedback on the Healthy Bone digital platform. A trained researcher (AM) conducted these sessions in Portuguese, which lasted approximately 70 minutes and were audio and screen recorded.

The Healthy Bone digital platform prototype was assessed through usability tests using a Think-Aloud approach [53]. This method was used to capture the end user’s thoughts as they interacted with the platform. First, participants were briefed on the study’s aims and introduced to the platform’s key features. They were then asked to complete several tasks with assistance from the moderator.

In the first task, participants had 5 minutes to explore the platform without specific instructions. After that, they were required to complete tasks that illustrated the use of the platform while verbalizing their thoughts. In task 2, patients were instructed to explore the main page, “My Program.” This entailed selecting and watching one of the available videos, clicking on the “Favorite” and “Like or Dislike” buttons, returning to the main page, toggling between the grid and slide views of the videos, and lastly, navigating to the “Favorites” page. In task 3, patients were asked to navigate to the “Questionnaires” page, select and complete one of the available weekly questionnaires, return to the “Questionnaires” page, and then select and complete one. In task 4, participants were instructed to navigate to the “Clinical Information” page. Task 5 required participants to explore the “Medication” page by clicking on it, visualizing the medication listed in the “Overview” tab, then going to the “Calendar” tab and checking all medications of that current day as taken. For task 6, participants explored the “Appointments” page, while in task 7, they had to navigate to the “Messages” page, write a message in the chat box, and send it. Finally, for task 8, patients only had to click on and investigate the “Profile” page.

After completing each task (except for the first task), participants were given an After Scenario Questionnaire (ASQ) [54] to measure task satisfaction. The ASQ includes three items with a 7-point scale, in which lower ratings represent a more favorable usability satisfaction of the system [54].

To assess the usability of the platform, and after carrying out all tasks, participants were asked to fill out:

1. System Usability Scale (SUS) [55]: SUS consists of 10 statements rated on a 5-point Likert scale of agreement strength. Scores range from 0 to 100 (higher scores representing higher usability) and can be interpreted by the grading scale proposed by [56] (eg, scores between 84.1 and 100 are an “A+”; between 80.8 and 84 are an “A”; between 78.9 and 80.7 are an “A-”; between 77.2 and 78.8 are a “B+”; etc). This scale is validated in the Portuguese population.
2. eHealth Usability Benchmarking Instrument (HUBBI) [46]: HUBBI is a new usability benchmarking tool that assesses usability in 7 domains: Basic System Performance, Task-Technology Fit, Interface Design, Navigation and Structure, Information and Terminology, Guidance & Support, and Satisfaction [46]. The authors of the HUBBI recommend using a radar chart to present the results, as these results are provided at a category level. A radar chart displays multivariate data in a 2-dimensional format, showcasing 3 or more quantitative variables represented on axes that originate from a common point. This view allows for a comprehensive overview of how each aspect of usability is performing [46].

After the usability tests, semistructured interviews were conducted to explore participants’ experiences with the Healthy Bone digital platform prototype, specifically their perceptions, the acceptability of the digital platform, usability issues, suggestions for improvement, and potential barriers and facilitators to use the digital platform. An interview guide was developed to conduct the interviews (Multimedia Appendix 1).

#### Data Analysis

This study followed a convergent mixed-methods approach, in which quantitative and qualitative data were collected in parallel, analyzed separately, and then integrated during interpretation. This process involved comparing findings and identifying convergences and divergences. For instance, SUS and HUBBI scores were subsequently analyzed alongside themes and usability issues emerging from the qualitative analysis to provide a more comprehensive understanding of the usability and user experience of the Healthy Bone digital platform.

#### Quantitative Analysis

Descriptive statistics were computed for sociodemographic and clinical characteristics, SUS, and HUBBI scores. Frequencies or proportions were used for categorical variables, means, and SDs were calculated for continuous variables. For the SUS, an overall score was calculated for each participant as previously described in the literature [57] so that final scores ranged from 0 to 100 (higher scores indicate better usability). All quantitative data were analyzed using SPSS software (version 29; IBM Corp) for macOS.

#### Qualitative Analysis

Before analyzing the qualitative data, the Think-Aloud usability tests and semistructured interview recordings were transcribed verbatim (AM) and anonymized with a pseudonym for each participant. The qualitative data analysis followed a deductive content analysis and inductive thematic analysis.

The deductive content analysis was performed using an ontology of usability problems [58] and was guided by the study’s aim of identifying potential usability issues. This analysis was specifically applied to the Think-Aloud approach. Usability issues were assigned as minor (occurring infrequently and/or slightly increasing task completion time), serious (occurring frequently and/or severely increasing task completion time), or critical (occurring universally among participants and/or preventing task completion), according to the severity index of Duh et al [59].

The identification of the usability issues and the coding of their severity were performed by one researcher (AM) through analysis of the transcripts and screen recordings. A second researcher (AB) reviewed the entire codification. Any disagreements were discussed and settled between the 2 researchers, and if a consensus could not be reached, a third researcher (AMR) was consulted. Following this, AM and AB independently classified each usability issue into one of the 8 categories and 21 factors of the ontology proposed by Broekhuis et al [58]. Once again, the 2 researchers (AM and AB) discussed the classifications to reach a consensus, with AMR intervening if necessary.

For the inductive thematic analysis, data coding was guided by the study’s aims of exploring patients’ use of the Healthy Bone digital platform prototype. This method allowed for a more open analysis of the semistructured interviews, with themes emerging directly from the data. This analysis followed the 6-phase process described by Braun and Clarke [60]: (1) familiarization with the data; (2) coding; (3) searching for themes; (4) reviewing themes; (5) defining and naming themes; (6) writing up. The entire process was independently performed by 2 researchers (AM and AB), who discussed the codes and themes that emerged from the analysis. When consensus on the coding could not be reached, a third researcher (AMR) was consulted to resolve the disagreement.

Given the small sample size and the combined inductive and deductive approach, we decided not to calculate formal interrater reliability statistics; instead, ensuring rigor through frequent and iterative discussions.

### Ethical Considerations

This study was conducted following the principles established by the Declaration of Helsinki, and ethical approval was granted by the NOVA Medical School|Faculdade de Ciências Médicas (NMS|FCM) Ethical Committee (CEFCM) (reference 196/2021/CEFCM). Eligible participants were provided with an information sheet detailing the purpose and procedures of the study. Those who agreed to participate received both written, patient-friendly materials and a verbal explanation of the study. Informed consent was obtained by the researcher, and all signed consent forms were securely stored in a location accessible only to the researchers. The procedures for obtaining informed consent were conducted in accordance with established ethical guidelines. The app is compliant with the European Union’s General Data Protection Regulation. All the collected data were anonymized. Compensation details were not applicable to this study.

## Results

### Sample Demographics

Seven patients participated in the study (mean 70.9, SD 6.2 years old; 5/7, 71.4% female). Most participants (5/7, 71.4%) reported owning and regularly using a computer, and all owned and used a smartphone. Among those who reported using a computer regularly, the most common task was browsing the internet (80%). Sociodemographic and clinical characteristics are presented in Table 1.

**Table 1.** Patients’ sociodemographic and clinical characteristics.

Variables	Total
Sex, n (%)	
Female	5 (71.4)
Male	2 (28.6)
Age, years	
Mean (SD)	70.9 (6.2)
Minimum	65
Maximum	81
Academic Qualification, n (%)	
Primary education (4 y of school)	1 (14.3)
Basic education (9 y of school)	1 (14.3)
Secondary education (12y of school)	2 (28.6)
Graduate degree	3 (42.9)
Use of technological devices^a^, n (%)	
Smartphone	7 (100)
Computer (desktop or laptop)	5 (71.4)
Tablet	4 (57.1)
Smartwatch	1 (14.3)
Most common tasks performed while using the computer, n (%)	
Working	2 (40)
Browsing the internet	4 (80)
Using email	1 (20)
Making videocalls	2 (40)
Sending messages	2 (40)
Browsing social media platforms	2 (40)
Functional disability (HAQ^b^), mean (SD)	0.3 (0.6)
Health-related quality of life (EQ-5D-3L)^c^, mean (SD)	0.8 (0.3)

a Technological devices add up to more than 7 since each participant may have more than one device.

b HAQ: Health Assessment Questionnaire.

c EQ-5D-3L: EuroQol, 5 dimensions, 3 levels.

### Usability Benchmarks

Participants reported high levels of usability on the SUS, with the Healthy Bone digital platform prototype achieving a mean overall SUS score of 87.1 (SD 13.3), corresponding to a grade of A+. This score exceeds the standard normal SUS distribution of 68 (SD 12.5) described in the literature as an important and useful benchmark [56]. The results of the calculated SUS scores are shown in Table 2.

**Table 2.** System Usability Scale scores per participant, total System Usability Scale score per patient, and total mean System Usability Scale score.

Participant ID	P1^a^	P2^a^	P3^a^	P4^a^	P5^a^	P6^a^	P7^a^	Mean SUS^b^ score (SD)
I think that I would like to use this system frequently	3	4	4	3	4	3	4	
I found the system unnecessarily complex	2	3	4	4	3	2	4	
I thought the system was easy to use	3	4	4	4	4	3	4	
I think that I would need the support of a technical person to be able to use this system	2	2	4	4	4	0	4	
I found the various functions in this system were well integrated	4	4	4	4	4	4	4	
I thought there was too much inconsistency in this system	3	4	4	4	4	4	4	
I would imagine that most people would learn to use this system very quickly	4	4	2	3	4	3	4	
I found the system very cumbersome to use	1	4	4	4	4	4	4	
I felt very confident using the system	2	4	4	3	4	2	4	
I needed to learn a lot of things before I could get going with this system	2	2	4	4	4	4	4	
SUS total score	65	87.5	95	92.5	97.5	72.5	100	87.1 (13.3)

a P1-P7: Patients 1-7

b SUS: System Usability Scale

**Figure 5.**
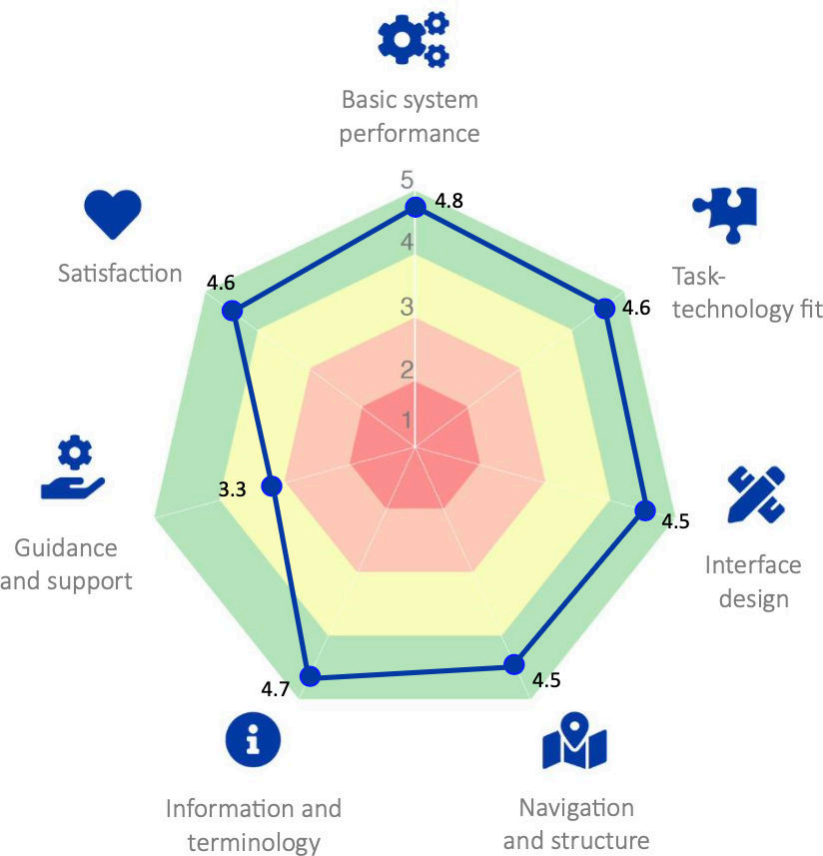
Visualization of the eHealth Usability Benchmarking Instrument scores for the Healthy Bone digital platform prototype. Each color indicates, for the different categories, how the system is performing. Green (>4) denotes good usability, yellow (3-4) moderate usability, orange (2-3) insufficient usability, and red (1-2) poor usability.

Regarding usability measured by the HUBBI, Figure 5 displays the average scores by category on a radar chart. The results indicate that the Healthy Bone digital platform prototype has good usability when considering the Basic System Performance (mean 4.8,SD 0.6), Task-Technology Fit (mean 4.6, SD 0.4), Interface Design (mean 4.5, SD 0.5), Navigation and Structure (mean 4.5, SD 0.7), Information and Terminology (mean 4.7, SD 0.4) and Satisfaction (mean 4.6, SD 0.4) categories. It can be observed that the scores for these categories are all placed within the green border of the radar chart. However, the digital platform prototype’s performance in the Guidance and Support category was only moderate (mean 3.3, SD 1.1) as evidenced by the yellow area of the radar chart.

Patients also reported high levels of task satisfaction, as measured by the ASQ (Table 3). Tasks 6 and 8 showed the highest score for task satisfaction (mean 1, SD 0). On the other hand, task 4 had the lowest task satisfaction (mean 1.8, SD 1.2).

**Table 3.** Task satisfaction scores are measured by the After Scenario Questionnaire.

Variables	Task 2	Task 3	Task 4	Task 5	Task 6	Task 7	Task 8
Mean (SD)	1.6 (0.9)	1.4 (0.7)	1.8 (1.2)	1.1 (0.3)	1 (0)	1.2 (0.4)	1(0)
Minimum	1	1	1	1	1	1	1
Maximum	3	2.7	4	1.7	1	2	1

### Usability Issues

In total, 48 usability issues were identified across all participants. After removing duplicates, 24 unique usability issues remained. On average, each participant encountered 3.3 (SD 2.5) unique minor usability issues, 2.1 (SD 1.6) unique serious usability issues, and 0.9 (SD 0.4) unique critical issues. The identified issues were classified into 6 usability categories and 10 usability factors. Table 4 describes all the usability issues identified in the analysis, along with the corresponding severity for each issue.

**Table 4.** Categorization of usability issues according to the eHealth usability ontology and the respective classification of their severity.

Categories, factors, and usability issues			Severity	Participants affected	First appearance
Basic system performance					
	Technical performance				
		When the user only checked one medication on a day with several medications to be taken, the corresponding day in the calendar turned green, when it should have turned yellow	Minor	P1	P1
		When clicking on the “Return” button inside a questionnaire, the system redirected the user to the “My Program” page instead of the main “Questionnaires” page.	Minor	P4	P4
		After completing a health-related self-reported questionnaire, the platform did not redirect the user to the “Concluded” section of the “Questionnaires” page; instead, it remained as if it had not been answered	Serious	P4; P5	P4
	General system interaction				
		The user tried to enter the platform without inputting the login code	Minor	P1; P2	P1
		The user had difficulty finding the “Pause” button when watching a video	Minor	P1; P6	P1
		The user had difficulty finding the “Return” button when inside a secondary page	Minor	P1	P1
		The user had difficulty distinguishing between clickable and nonclickable elements	Serious	P1; P3; P4	P1
		The user did not know how to move the analog scale within the EQ-5D-3L questionnaire (they were unaware they needed to click, hold, and slide the scale)	Minor	P1	P1
		The user did not know how to conclude a patient-reported outcome measure	Minor	P2	P2
		The user had difficulty sliding the videos when in list view (“My Program” page)	Minor	P4	P4
		The user had difficulty finding the “Calendar” button in the “Medication” page	Minor	P7	P7
Accessibility					
	Accommodation to perceptual impairments or limitations				
		The user had difficulty seeing some elements of the platform, due to their small size and their vision problems	Minor	P1	P1
Interface design					
	Design clarity				
		The user interpreted the image of a heart used to represent “Other medication” as indicating cardiac medication	Minor	P1; P5	P1
		The user had difficulty noticing if the “Favorite” and “Like/ dislike” buttons were selected or not	Minor	P1	P1
		The user had difficulty finding the “Favorite” and “Like/ dislike” buttons due to their small size	Minor	P4; P7	P4
		The user had difficulty differentiating between weekly questionnaires and health-related self-reported questionnaires in the “Questionnaires” page	Minor	P4	P4
	Symbols, icons, and buttons				
		The user did not know how to switch between list and grid view and which button to use	Serious	P1; P4; P6	P1
Navigation and Structure					
	Navigation				
		The user did not know how to navigate from the “My Program” main page to the “Favorites” page	Minor	P4	P4
	Structure				
		The user had difficulty understanding the weekly questionnaires component and what they were supposed to do	Minor	P2	P2
		The user did not understand the graphics shown in the “Clinical information” page and their relationship with the health-related self-reported questionnaires	Serious	P2; P3; P5 P6	P2
		The user did not understand the “Participation in the program” section of the “Profile” page	Serious	P3; P4; P5	P3
Information and terminology					
	Health-related information				
		The user did not understand the use of the terms “Quantity”, “Frequency” and “Duration” in the “Overview” section of the “Medication” page.	Serious	P1; P4; P6	P1
Guidance and support					
	Error management				
		The user did not know they could change the answers to the weekly questionnaires when an incorrect answer was chosen as the system did not provide this information	Critical	P2; P3; P4 P5; P6; P7	P2
	Procedural health-related information				
		The user did not know how and where to log their medication as taken	Minor	P6	P6

Usability issues were identified within both factors of the Basic system performance usability category. In the Technical performance factor, 3 (12.5%) issues were found, such as, “When clicking on the ‘Return’ button inside a questionnaire, the system redirected the user to the ‘My Program’ page instead of the main ‘Questionnaires’ page.” In the General system interaction factor, 8 (33.3%) issues were identified, including problems like “The user had difficulty distinguishing between clickable and non-clickable elements” or “The user did not know how to conclude a patient-reported outcome measure.”

In the Accessibility category, only 1 (4.2%) issue was identified and classified within the Accommodation to perceptual impairments or limitations factor: “The user had difficulty seeing some elements of the platform due to their small size and their vision problems.” In the Interface Design category, 4 (16.7%) issues were found under the Design clarity factor (eg, “The user had difficulty noticing if the “Favorite” and “Like or Dislike” buttons were selected or not”), and only 1 (4.2%) issue was found in the Symbols, icons, and buttons factor (ie, “The user did not know how to switch between list and grid view and which button to use”).

In the Navigation and Structure category, usability issues were identified in both related factors. In the Navigation factor, only 1 (4.2%) issue was found: “The user did not know how to navigate from the ‘My Program’ main page to the ’Favorites’ page.” In the Structure factor, 3 (12.5%) issues were identified, such as “The user had difficulty understanding the weekly questionnaires component and what they were supposed to do.”

In the Information and terminology category, 1 (4.2%) usability issue was found in the Health-related information factor: “The user did not understand the use of the terms ‘Quantity’, ‘Frequency’, and ‘Duration’ in the ‘Overview’ section of the ‘Medication’ page.” Finally, usability issues were also identified in the category of Guidance and support. The analysis found 1 (4.2%) issue in the Error management factor (ie, “The user did not know they could change the answers to the weekly questionnaires when an incorrect answer was chosen as the system did not provide this information”) and 1 (4.2%) other issue in the Procedural health-related information factor (ie, “The user did not know how and where to log their medication as taken”).

### Semistructured Interviews

#### Acceptability and Perceived Usefulness of the Healthy Bone Platform

The patients generally described Healthy Bone as an easy-to-use, accessible, and visually appealing platform. They expressed satisfaction with its simplicity and the way the platform was organized. In addition, they considered that the platform had the potential to empower individuals with osteoporosis, helping them better manage the disease and improve their overall quality of life.

*I’m pleased with the platform as a whole, the way it is presented, its simplicity in terms of the tasks, the videos… simple and easy to understand. Then… the different pages of the menu, they also seem relatively easy to access, I liked it, yes.*

[P4]

*Because this gives us tips for our daily lives and I think this is important for anyone, right? Especially those who have this health problem [osteoporosis] […] Sometimes we don't have anyone to give us advice on something, and we can come here and see it, right?*

[P5]

Despite this, some patients noted that some people may experience difficulties interacting with the platform, and an adaptation period could be necessary. This view was mainly explained by the fact that the platform requires a minimum set of digital skills, which may not be common among many older adults. However, the platform’s simplicity and the support from family members were pointed out as potential factors that could help overcome this issue.

*I think it’s simple, but I can't say with 100% certainty that a person who is not as used to, or who only uses a computer once in a while… there’s always some degree of difficulty at the beginning […*]

[P4]

*On a technological level, exactly. I think it is necessary to have a minimum level of knowledge on computers to… from a user’s perspective because the program is very… it presents itself with an enormous simplicity.*

[P7]

Overall, the patients found the different components of the Healthy Bone digital platform acceptable and useful. Patients thought that the available videos discussed important topics related to osteoporosis and general health. They also considered the “Medication” and “Clinical Information” pages important assets as they facilitated better monitoring of medication and health outcomes, respectively. In addition, patients were pleased with the “Messages” section of the Healthy Bone platform. Aspects, such as the possibility of having a closer connection to their respective doctors and being able to communicate with them easily, were regarded as advantages of the platform, transmitting tranquility and greater confidence. The reminders feature was also seen as essential, serving as an important alert for patients, reminding them about upcoming appointments, medication schedules, or even informing them of new video releases.

*Look, regardless of osteoporosis, I take other medications, and I believe that, when this is ready and if I have access to this platform… that it will be able to help me, right?*

[P1]

*[…] that makes sense because there is a closer connection between us and our doctor, and knowing that at any moment I can communicate… I may not have an immediate answer, but I will have it […] it offers a kind of tranquillity to the patient and a little more confidence […*]

[P4]

#### Barriers to Using the Healthy Bone Platform

Patients reported potential barriers that may hamper the use of the Healthy Bone platform. The main barrier, reported by almost all patients, was a lack of skills and knowledge in using digital technologies. Even though the platform was regarded as simple, easy, and accessible, it was noted that people with limited digital skills could have more difficulty engaging with the system. Some patients reported not being used to using digital technologies, especially computers, and having lost skills over time due to disuse.

*Well, ok… for me, I honestly have some difficulty. I don't know… there are people of my age with more skills for these technologies, which is not very much my case. I always ran away from computers, and now I am… well, a layperson.*

[P1]

*The lack of basic knowledge […] to work with, with the application. Knowledge from, from a user’s perspective […*]

[P7]

*[…] when I was working, I had a lot of contact with… it wasn't these technologies that we have today, obviously. It was much more… things were less advanced, but I worked with computers, all those things. However, from the moment that I stopped working and stayed home with my husband, both of us stayed home, things got lost, got a little lost.*

[P6]

The lack of suitable equipment, such as computers and smartphones, was also a barrier to actively using the platform and participating in the program.

*Well, there are people who prefer their smartphone or a tablet and others to a computer, but really, the biggest barrier will be the lack of equipment.*

[P7]

Other barriers were also reported by patients, such as having a busy life and not having the time to access the platform, as using the platform may imply daily access and a higher commitment. Some expressed concerns about not enjoying using digital technologies.

*Sometimes the lack of time, for instance […] I don’t know, people sometimes are so… we’re all so busy, right? It’s not just some people, it’s everyone. And sometimes there may be a lack of time to go… to use the platform […*]

[P5]

#### Facilitators Using the Healthy Bone Platform

While a lack of equipment was identified as a barrier, having access to the necessary equipment and being comfortable with digital technologies were viewed as potential facilitators for using the Healthy Bone platform. Patients also highlighted the importance of expecting potential positive benefits from the program, which could motivate them to engage with the system. In addition, a desire to improve overall health and adopt healthy behaviors was identified as a key factor in promoting regular use of the platform.

*[…] people have to want to improve their lifestyle, want to improve, want to have better health results […] If the person doesn’t want that […] that’s the first, in my opinion. The second is, is to have the necessary equipment at your disposal […*]

[P7]

*For many of the things I’ve already said, because I’m interested in improving my quality of life, in losing some weight, and because I want to get up without pain in my joints, what else? For everything, for everything.*

[P7]

*Well… people also need to be predisposed to do it, right? And be interested in what they’re doing, if they’re doing it well, if not, sometimes people don’t have anyone to advise them, and so they can see on those graphs how they’re progressing, how they’re doing things (…) The person also needs to be willing to use a little bit of their time to, to interact.*

[P5]

Access to important and accurate information about the disease, along with strategies to improve health and promote behavior change, and the ability to visualize their improvements and program progression were also highlighted as facilitators.

*That thing of having a quick overview, at the moment, of how you are doing, of your evolution in terms of adherence to physical activity, the suggestions for changes in eating behaviors, the progress in terms of using the platform, and your evolution in terms of healthy lifestyle habits.*

[P4]

In addition, the ability to access the platform through a smartphone or a tablet app was seen as a facilitating factor. This was primarily because older adults are generally more familiar with smartphones than computers, making them better adapted to smartphones.

*It depends (…) it depends on each person’s level of education and whether or not they are used to their smartphone. Because on a smartphone, it won't be like that. It will be with your finger, right? […] I don’t know, I don’t know, but… it just might be easier on a smartphone or tablet*.

[P3]

This platform was also found to have an important impact on individuals living in geographically remote areas, as it allowed them to receive care despite their location, thus decreasing access inequalities.

*[…] look, I live 30 or so kilometers away, around 40. If I must call, there are difficulties, difficulties in terms of service provision […] And this, this is the best way to communicate, I see that… I had an aversion to new technologies. I was against them, and now I’m paying dearly for that […] a lot of things can be solved with this type of digital technologies.*

[P1]

## Discussion

### Principal Findings

This study provides a comprehensive assessment of the usability and user experience of the desktop version of the Healthy Bone digital platform prototype from the perspective of participants with osteoporosis. The mixed-methods methodology enabled the collection of valuable information on the platform’s usability, as well as users’ experiences and perceptions. These findings will be crucial for optimizing the current version of the prototype.

High levels of usability of the Healthy Bone digital platform were found in this study. The mean overall SUS score was 87.1, corresponding to a grade of A+, which indicates excellent usability. High usability was also found when considering the HUBBI individual category scores. The system scored moderately (mean 3.3, SD 1.1) in the Guidance and Support category, indicating that the platform’s features related to this category must be improved to enhance usability. This lower score could be explained by specific HUBBI items (eg, items 14 and 15) that focus on error management and procedural system information, namely providing information on how to perform several tasks within the system (eg, creating an account, logging in, and changing settings) [46]. At the time of the usability testing, the Healthy Bone digital platform was considered a minimum viable product. It was at a stage suitable for potential end-users but not yet finalized. Therefore, features such as error notifications were not implemented. Nonetheless, this represents valuable information that indicates that adding these features to the platform could significantly improve its usability. In addition, the Healthy Bone digital platform will require training to ensure patients know how to perform several tasks related to system procedures. This training can be administered by their assisting health care professional or other competent professionals according to where the Healthy Bone platform is first presented to the patient.

The use of both the SUS and the HUBBI represents a methodological strength, as it allows for a more comprehensive assessment of usability and user experience. One common criticism of the SUS is its limited ability to offer detailed insights into which aspects of digital technologies need to be refined or modified. In contrast, the HUBBI, which was developed based on the eHealth ontology of usability issues, addresses this gap by providing scores for each relevant category of usability instead of a single total score [46]. This allows for a more comprehensive assessment of how digital innovation is performing and highlights the aspects that researchers and designers should focus on during the optimization phase of development.

However, this approach also introduces some trade-offs. Specifically, administering multiple scales can increase the burden on participants, particularly in clinical populations who may already experience cognitive or physical fatigue. In addition, interpreting results across different instruments can add complexity to the analysis, particularly when findings are not fully aligned. These challenges highlight the need for careful planning in future studies to balance depth of insight with participant engagement and analytical clarity.

The qualitative data further supported the quantitative results regarding the usability of the Healthy Bone digital platform. The Think-Aloud approach provided an opportunity to observe how end-users interacted with different components of the system while performing relevant tasks that simulated real-world usage of the digital platform. This process was crucial and helped identify specific usability issues affecting the platform’s use, which could be addressed and improved. The analysis of the usability issues was guided by the eHealth ontology [58], which was the basis for developing the HUBBI. This approach enabled a deeper exploration of the HUBBI constructs, providing a better understanding of specific issues that could affect usability within each HUBBI category. While several usability issues were identified, most were classified as minor or serious, indicating that they occurred infrequently or frequently among patients, respectively. However, none of these issues obstructed patients from completing the required task.

No usability issues were found within the categories of Task-technology fit (ie, usability problems that occur because the user does not consider that the system is suitable for meeting their needs or goals) and Satisfaction (ie, the user’s satisfaction with the system and their subjective opinion regarding the system’s likeability). Some quantitative and qualitative analysis results can explain this absence. Patients reported high levels of task satisfaction, and the qualitative interview results further supported this. Participants consistently expressed satisfaction with the simplicity, accessibility, and utility of the different components of the Healthy Bone digital platform. These positive views suggest high satisfaction levels and the platform effectively aligns with patients’ needs. However, one concern that emerged was the level of digital skills required to engage with the system. Some participants indicated that certain older adults may have limited technological proficiency and, therefore, more difficulties using the Healthy Bone digital platform, impacting its acceptability.

The patients also identified other barriers and facilitators to using the Healthy Bone platform. The main reported barriers were a lack of digital skills, unsuitable equipment, and time constraints due to a busy life, which might compromise the platform’s adoption. On the other hand, patients highlighted several facilitators that could promote engagement and adoption of the Healthy Bone platform. These included having access to adequate equipment, being motivated to change behaviors, experiencing important health benefits, and decreasing potential inequalities.

The findings regarding limited access to ICTs and the lack of skills to effectively engage with them align with the current literature on the “digital divide.” This term describes the gap between individuals with sufficient access to and proficiency using ICTs and those who lack these resources and skills [61,62]. Recent evidence suggests that this digital divide is growing, particularly among older adults, potentially affecting the quality and effectiveness of the health care they receive [62]. Bridging this digital divide and promoting the use of ICTs should be a priority, as it has the potential to contribute to achieving the United Nations’ Sustainable Development Goal 10, reducing inequality within and among countries [63,64].

The convergent mixed-methods analysis allowed the identification of clear points of convergence between usability metrics and user experience narratives, as well as specific gaps that qualitative findings helped explain. Generally, the high SUS score of 87.1 was consistent with participants’ reports of the platform being a simple, easy-to-learn, and visually appealing tool. These perceptions also align with stronger HUBBI domains related to the overall interface quality and satisfaction of the digital tool. On the other hand, weaker HUBBI domains, such as guidance and support, reflected user concerns about lacking procedural instructions. Importantly, the only critical usability issue (ie, not knowing they could change the answers to the weekly questionnaires when an incorrect answer was chosen as the system did not provide this information) was mapped to this specific domain.

### Comparison With Prior Work

Previous studies have also examined barriers and facilitators to the use and adoption of eHealth solutions [65,66]. Interestingly, barriers and facilitators to the use of the Healthy Bone digital platform were in line with some of the most reported factors in the literature. A systematic review by Schreiweis et al [65] identified factors such as poor digital health literacy, lack of the necessary devices, and the added workload as some of the most common barriers to using eHealth services. The review also found that the ease of use, communication improvements, and availability of resources constituted some of the most reported enablers.

The findings of this study will significantly contribute to optimizing the current prototype of the Healthy Bone digital platform. Several strategies will be used, encompassing the correction of technical performance issues, modifications to the platform (eg, increasing the font size, increasing the visibility and clarity of buttons to make them more easily detectable within the interface), and developing a user guide that provides clear explanations on how to interact with the different components of the platform.

This study may also have important implications for developing and implementing eHealth solutions aimed at preventing and managing osteoporosis-related fractures. Specifically, it provides comprehensive information regarding the facilitators, barriers, and usability issues that may be common to several digital solutions for patients with osteoporosis or osteoporosis-related fractures.

This Healthy Bone digital platform includes a multifactorial innovative intervention, allowing it to address health monitoring, including health literacy, and support adherence from a distance in the older adults, a vulnerable group, as we found with the COVID-19 experience. This project intends to add value to the knowledge of how these types of ICT tools can be used in supporting osteoporosis patients.

### Limitations

This study has some limitations that must be addressed. The SUS mean score of 87.1 suggests high levels of usability; however, the small sample size may affect the generalizability of the findings. Although evidence suggests that around 80% of usability issues can be found with just five users [48,67,68], larger and more diverse samples are needed to confirm these findings. Although our sample size aligns with usability testing norms, we acknowledge that full thematic saturation may not have been achieved. Two new usability issues were identified in the analysis of the last 2 interview transcripts, and the small sample size limits the certainty regarding the saturation of the acceptability and usefulness, barriers, and facilitators findings. A larger and more diverse sample size could have provided more robust data, and future studies are needed to expand upon these findings. Another limitation of this study concerns the scope and setting of the usability testing. First, it is the fact that usability testing was restricted to the desktop version of the Healthy Bone platform, as the mobile and smart TV versions were still under development. This hampers the generalizability of our findings, particularly considering that older adults may find it preferable to access digital solutions via other devices other than computers. Second, all usability tests were conducted in a controlled, research-led environment, which may have increased perceived usability. Future research on this project should include usability testing of the other versions of the digital platform, ensuring that the platform is well adapted across all devices, and include home-based, independent testing to capture a more comprehensive overview of usability in real-world conditions.

Furthermore, patients who regularly use smartphones and health management apps may have been more inclined to participate, which could introduce a bias toward higher eHealth literacy, acceptability, and satisfaction with the digital platform.

### Conclusions

This study has provided valuable insights into the usability and user experience of the desktop version of the Healthy Bone digital platform prototype. Specific usability issues and users’ perceptions of this digital solution were identified. The next steps will involve evaluating the usability and user experience of the mobile app version of Healthy Bone, followed by a pilot study and a randomized controlled trial. Data gathered from the usability and user experience assessments will ensure that the digital solution is tailored to the needs of the target population. This knowledge can lead to a more successful implementation and maximize the overall impact of the Healthy Bone digital platform.

## Supplementary material

10.2196/72468 Multimedia Appendix 1 Semistructured interview guide.

10.2196/72468 Checklist 1 GRAMMS checklist.

## Abbreviations

API: Application Programming Interface
ASQ: After Scenario Questionnaire
CMS: Content Management System
EQ-5D-3L: EuroQol, 5 dimensions, 3 levels
GRAMMS: Good Reporting of a Mixed Methods Study
HAQ: Health Assessment Questionnaire
HUBBI: eHealth Usability Benchmarking Instrument
ICT: information and communication technology
SUS: System Usability Scale
