# Supplementary material for: Usability and User Experience of a Digital Platform Prototype (Healthy Bone) to Promote Pharmacological and Nonpharmacological Treatment in Patients With Osteoporosis: Mixed Methods Study
Source: JMIR Form Res. 2025 Nov 7;9:e72468. doi: 10.2196/72468 (PMC12594503; doi:10.2196/72468)
Supplement: Multimedia Appendix 1 [file formative-v9-e72468-s001.pdf]

Semi-structured interview guide

- What was your first impression of the Healthy Bone digital platform?
- How was your user experience?
  - Did you find the platform easy to use? Was it easy to complete the requested tasks?
  - Did you find the information on the platform readable and understandable?
  - Positive aspects/advantages and negative aspects/disadvantages of its use?
  - Were there any interactions with the app that you found frustrating?
  - Are there a screens with a lot of information?
  - Do you think the platform is suitable for older people (≥65 years)? In what way?
- What barriers, if any, do you feel might prevent people from using the Healthy Bone platform?
- What facilitators, if any, do you feel can help people use the HealthyBone platform?
- What do you think about the length of the videos?
- What do you think of the several components of the Healthy Bone platform?
- What do you think about the total duration of the intervention (6 months of new content + 6 months of personalized content according to the needs of each person)?
- In the future, would you use the Healthy Bone platform? If so, why? If not, why?
  - Do you think you would like to use this platform frequently?
  - Are there any aspects that you think are missing from the Healthy Bone platform?
- Is there anything you would like to say or add about this part of the Healthy Bone prototype that you just saw?
- What suggestions/recommendations would you give to improve the Healthy Bone platform?
