# Supplementary material for: Usability and User Experience of a Digital Platform Prototype (Healthy Bone) to Promote Pharmacological and Nonpharmacological Treatment in Patients With Osteoporosis: Mixed Methods Study
Source: JMIR Form Res. 2025 Nov 7;9:e72468. doi: 10.2196/72468 (PMC12594503; doi:10.2196/72468)
Supplement: Checklist 1 [file formative-v9-e72468-s002.pdf]

Good Reporting of a Mixed Methods Study (GRAMMS) checklist

**Guideline**	**Section: Page**
Describe the justification for using a mixed methods approach to the research question	Methods - Data analysis: Page 14
Describe the design in terms of the purpose, priority and sequence of methods	Methods – Study design: Page 7<br>Methods – Data Collection and Data Analysis: Pages 12-15
Describe each method in terms of sampling, data collection and analysis	Methods – Participants and recruitment: Page 7<br>Methods – Data Collection and Data Analysis: Pages 12-15
Describe where integration has occurred, how it has occurred and who has participated in it	Methods – Data analysis: Pages 14-15
Describe any limitation of one method associated with the present of the other method	Discussion – Limitations: Pages 29-30
Describe any insights gained from mixing or integrating methods	Discussion: Pages 26 - 28

O'cathain Alicia, Murphy Elizabeth, Nicholl Jon. The Quality of Mixed Methods Studies in Health Services Research. J Health Serv Res Policy SAGE Publications; 2008 Apr 1;13(2):92–98. doi: 10.1258/jhsrp.2007.007074
